# Asymmetric Patterns of Small Molecule Transport After Nanosecond and Microsecond Electropermeabilization

**DOI:** 10.1007/s00232-017-9962-1

**Published:** 2017-05-08

**Authors:** Esin B. Sözer, C. Florencia Pocetti, P. Thomas Vernier

**Affiliations:** 1 Frank Reidy Research Center for Bioelectrics, Old Dominion University, 4211 Monarch Way, Ste. 300, Norfolk, VA 23508 USA; 2 Department of Bioengineering, Instituto Tecnológico de Buenos Aires, Buenos Aires, Argentina

**Keywords:** Electroporation, Asymmetric molecular transport pattern, Nanosecond electropermeabilization

## Abstract

Imaging of fluorescent small molecule transport into electropermeabilized cells reveals polarized patterns of entry, which must reflect in some way the mechanisms of the migration of these molecules across the compromised membrane barrier. In some reports, transport occurs primarily across the areas of the membrane nearest the positive electrode (anode), but in others cathode-facing entry dominates. Here we compare YO-PRO-1, propidium, and calcein uptake into U-937 cells after nanosecond (6 ns) and microsecond (220 µs) electric pulse exposures. Each of the three dyes exhibits a different pattern. Calcein shows no preference for anode- or cathode-facing entry that is detectable with our measurement system. Immediately after a microsecond pulse, YO-PRO-1 and propidium enter the cell roughly equally from the positive and negative poles, but transport through the cathode-facing side dominates in less than 1 s. After nanosecond pulse permeabilization, YO-PRO-1 and propidium enter primarily on the anode-facing side of the cell.

**Electronic supplementary material:**

The online version of this article (doi:10.1007/s00232-017-9962-1) contains supplementary material, which is available to authorized users.

## Introduction

Electropulsation-facilitated small molecule uptake is widely used in biomedical and industrial applications like electrochemotherapy (Marty et al. 2006), calcium electroporation (Frandsen et al. 2012), and food processing (Dymek et al. 2014). Decades of investigations have provided insights into the process (Teissie et al. 2005; Teissie 2014), including details of electropore formation in lipid bilayers (Tieleman 2004; Delemotte and Tarek 2012), but our knowledge of the mechanisms of the transport of normally impermeant small molecules across electropermeabilized membranes remains schematic and hypothetical. Although models of electroporation have evolved in response to the accumulation of experimental data over the years (Neu and Krassowska 1999; Son et al. 2014), their ability to predict a variety of experimental outcomes is limited.

For example, the electro-physics-based “standard model” of electroporation describes the transmembrane potential induced by an external pulsed electric field as a function of position on the membrane (confirmed by experiments (Hibino et al. 1991, 1993), but fails to predict the patterns of entry of charged, impermeant small molecules into electropermeabilized cells. In the model, the probability and extent of electropore formation depend directly on the magnitude of the transmembrane potential, so we expect that the sites of transport and the amount of material transported will be correlated with the transmembrane potential distribution over the surface of the cell. Because the transmembrane potential induced by an external electric field is greatest at the poles of the cells (along an axis aligned with the direction of the electric field) (von Pauly and Schwan 1959), the model leads us logically to predict more pore formation (and therefore more transport) at the poles, and a somewhat greater effect at the anode (positive electrode)-facing pole than at the cathode side, because of the summing of the induced and resting transmembrane potentials (Tekle et al. 1990; DeBruin and Krassowska 1999).

What is actually observed is that in some cases (Tekle et al. 1990; Tekle et al. 1994; Mehrle et al. 1985, 1989) transport occurs more on the *anode*-facing regions of the membrane, while in others (Sowers and Lieber 1986; Dimitrov and Sowers 1990; Kinosita et al. 1992; Tekle et al. 1994) transport is greater on the *cathode* side. Later studies using fast imaging technology report *anode*-side permeabilization when the observations are made in the first few seconds after pulse exposure (Gabriel and Teissié 1997, 1999; Sadik et al. 2013). To explain this, Tekle et al. (1994), who observed *anode*-side entry for calcium and ethidium ions but *cathode*-side entry for propidium and ethidium homodimer, proposed that a larger number of smaller pores form on the anode side (allowing the smaller Ca^2+^ and ethidium to enter preferentially over the larger propidium and ethidium homodimer), and a smaller number of larger pores form on the cathode side. Some models are consistent with this idea (Krassowska and Filev 2007, Saulis 1993). An alternative hypothetical mechanism for cathode-dominant transport is electro-osmosis (Dimitrov and Sowers 1990).

The studies referenced above all involve pulses in the microsecond or millisecond range. For cases of nanosecond pulse permeabilization, one report of preferential entry of YO-PRO-1 through the anode-facing side of cells (Vernier et al. 2006a) was attributed to the unequal transmembrane potentials on the two hemispheres of the cell (anode-side potential greater than cathode-side) that result from superposition of the resting potential with the potential induced by the pulsed external electric field (Pakhomov et al. 2009). To date there have been no reports of cathode-side entry following nanosecond electropermeabilization, to our knowledge. One model of nanosecond electroporation suggests that pore formation following a single-pulse exposure occurs everywhere on the membrane, with no preferential site (Gowrishankar et al. 2006; Son et al., JMB 2014). Consistent with this prediction, global entry of Ca^2+^ was observed following 10 ns pulse exposures, while in the same system, a 4 ms pulse caused polarized (anode and cathode) Ca^2+^ influx (Semenov et al. 2015).

We report here patterns of entry of YO-PRO-1, propidium, and calcein—impermeant small molecules commonly used as indicators of membrane permeabilization—into U-937 lymphoid cells following exposure to nanosecond and microsecond permeabilizing pulsed electric fields. In microsecond pulse experiments (1, 220 µs pulse), *cathode*-side entry of YO-PRO-1 and propidium dominates within 1 s after the pulse. In nanosecond multiple-pulse experiments (10, 6 ns pulses), we observe a preferential, *anode*-side entry for YO-PRO-1 and propidium that intensifies with increasing pulse repetition rate. YO-PRO-1 influx following a single 6 ns pulse is nearly symmetric around the circumference of the cell. There is no strong anode- or cathode-side preference in the first few seconds, but over 2 min the anode-side fluorescence increases slightly more than the cathode-side. Only very low levels of calcein entry are detected under our measurement conditions, with no discernible preference for anode or cathode side.

## Materials and Methods

### Cells

U-937 (human histiocytic lymphoma monocyte; ATCC CRL-1593.2) cells (Sundström and Nilsson 1976) were cultured in RPMI-1640 medium (Corning^®^ glutagro™ 10-104-CV) with 10% fetal bovine serum (Corning, 35-010-CV) and 1% penicillin/streptomycin (10,000 U mL^−1^ penicillin and 10 mg mL^−1^ streptomycin) at 37 °C in a humidified, 5% CO_2_ atmosphere.

### Pulsed Electric Field Exposure

**Fig. 1:**
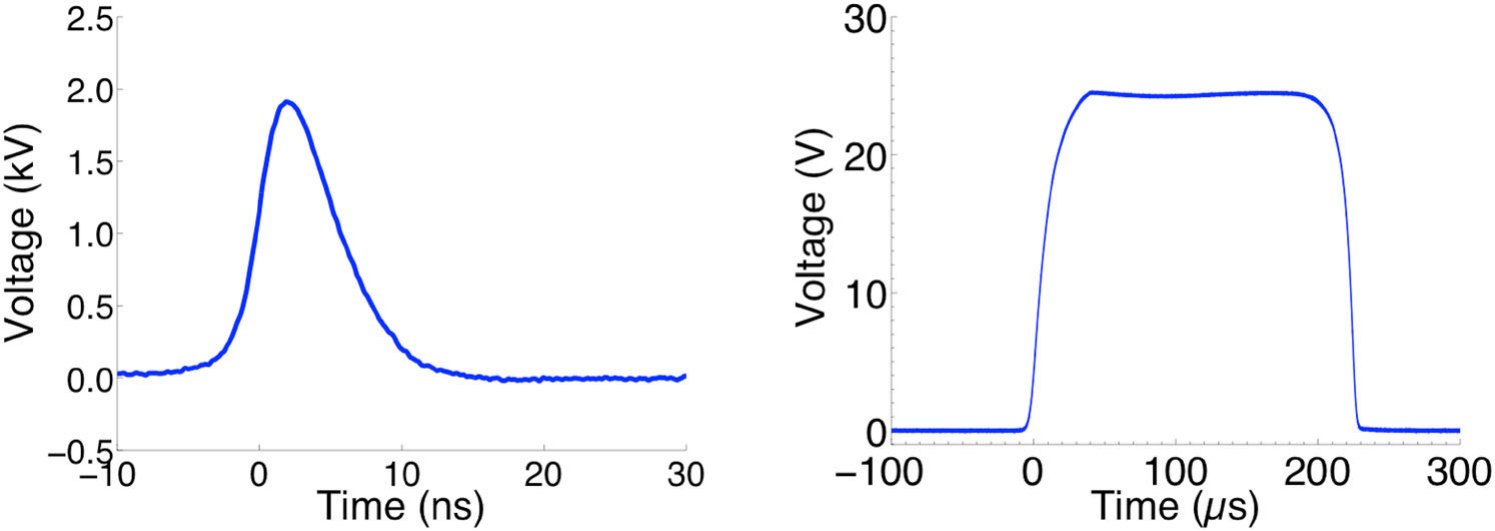
Typical 6 ns and 220 µs waveforms recorded during application to experimental samples

6 ns, 20 MV m^−1^ pulses (FID pulse generator FPG 10-10NK) or 220 µs, 250 kV m^−1^ pulses were delivered to cells in suspension in cover glass chambers [Nunc™ Lab-Tek™ II] through parallel tungsten wire electrodes with 100 µm gap (Wu et al. 2013). Cells were observed at laboratory temperature on the stage of a Leica TCS SP8 laser scanning confocal microscope. Waveforms can be seen in Fig. 1.

### Molecular Transport

Cells were washed and suspended at approximately 5 × 10^5^ cells mL^−1^ in fresh medium containing either 2 µM YO-PRO-1, 30 µM propidium, or 200 µM calcein. At least 30 cells from three independent experiments are analyzed for each reported dataset unless otherwise indicated.

### Imaging

Laser scanning confocal fluorescence microscope images were captured (Leica TCS SP8) every 100 ms (YO-PRO-1 and propidium) or every 200 ms (calcein) for 2 min (1200/600 frames) from cell suspensions at room temperature in ambient atmosphere on the microscope stage. Cells were exposed to electrical pulses five seconds after the start of the recording.

### Image Processing

**Fig. 2:**
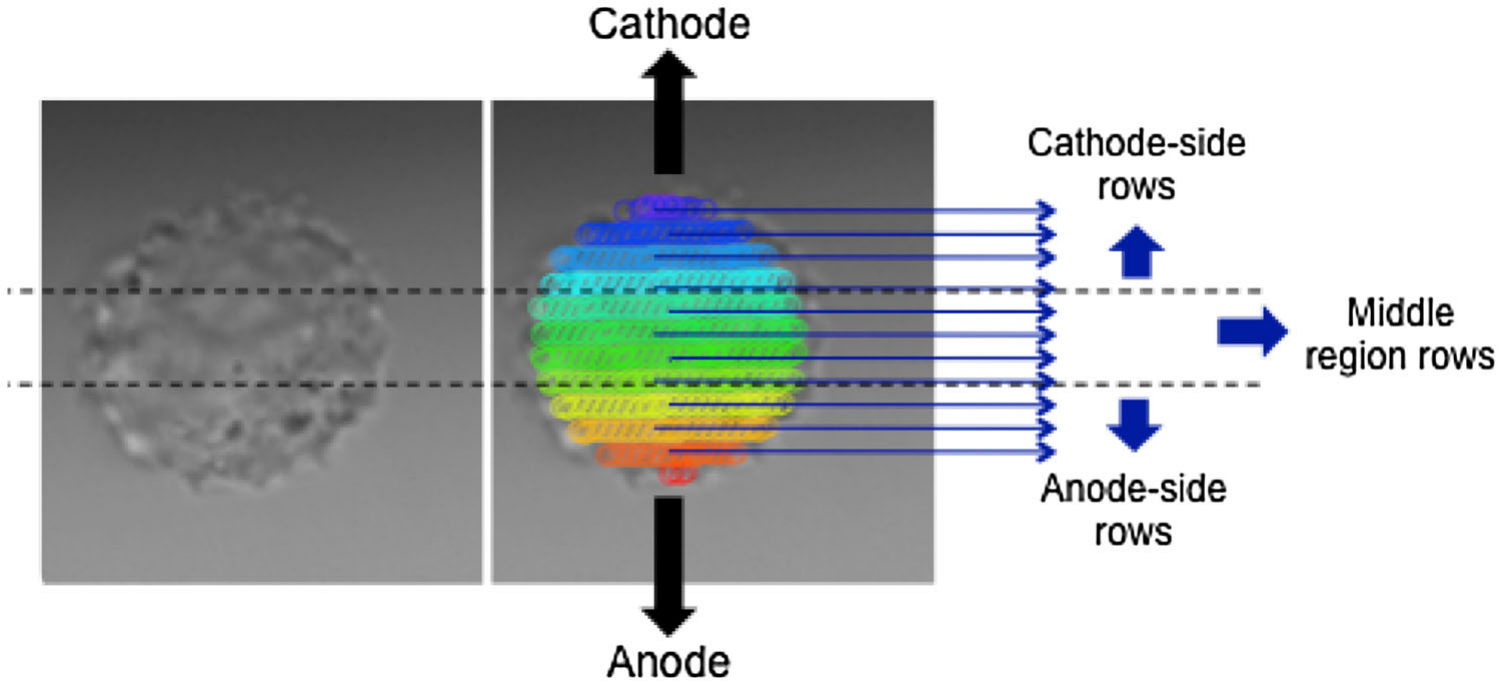
Pattern analysis of a single cell. The cell area is divided into rows of pixels (represented schematically by the regions of *different colors*; in the actual analysis, there are roughly 50 rows of pixels per cell) parallel to the electrodes. The rows are grouped into cathode- and anode-side and middle regions and the average of the mean intensities of the rows is reported as the intensity of the region (Color figure online)

All cells visible in the microscope field between the electrodes were manually selected for fluorescence photometric image analysis before each pulse exposure. Fluorescence intensities of each region of interest were extracted using custom MATLAB routines that allow tracking of cells in different frames. The following built-in MATLAB functions were used in custom image processing routines.: ‘imroi,’ for manually choosing regions of interest; ‘regionprops,’ for evaluating geometric properties of regions of interest. For the pattern analysis of molecular uptake, the cell area, individually for each cell, was divided into rows of pixels aligned in parallel with the electrodes, and the mean fluorescence intensity of each row was calculated. The rows were partitioned into three groups (anode-facing, middle, and cathode-facing) with each group containing one-third of the total rows. The intensity reported for each group is the average of the mean intensities of the rows within that group (Fig. 2). When plots are indicated as smoothed, a second-order Savitzky–Golay filter was used.

### Calibration

**Fig. 3:**
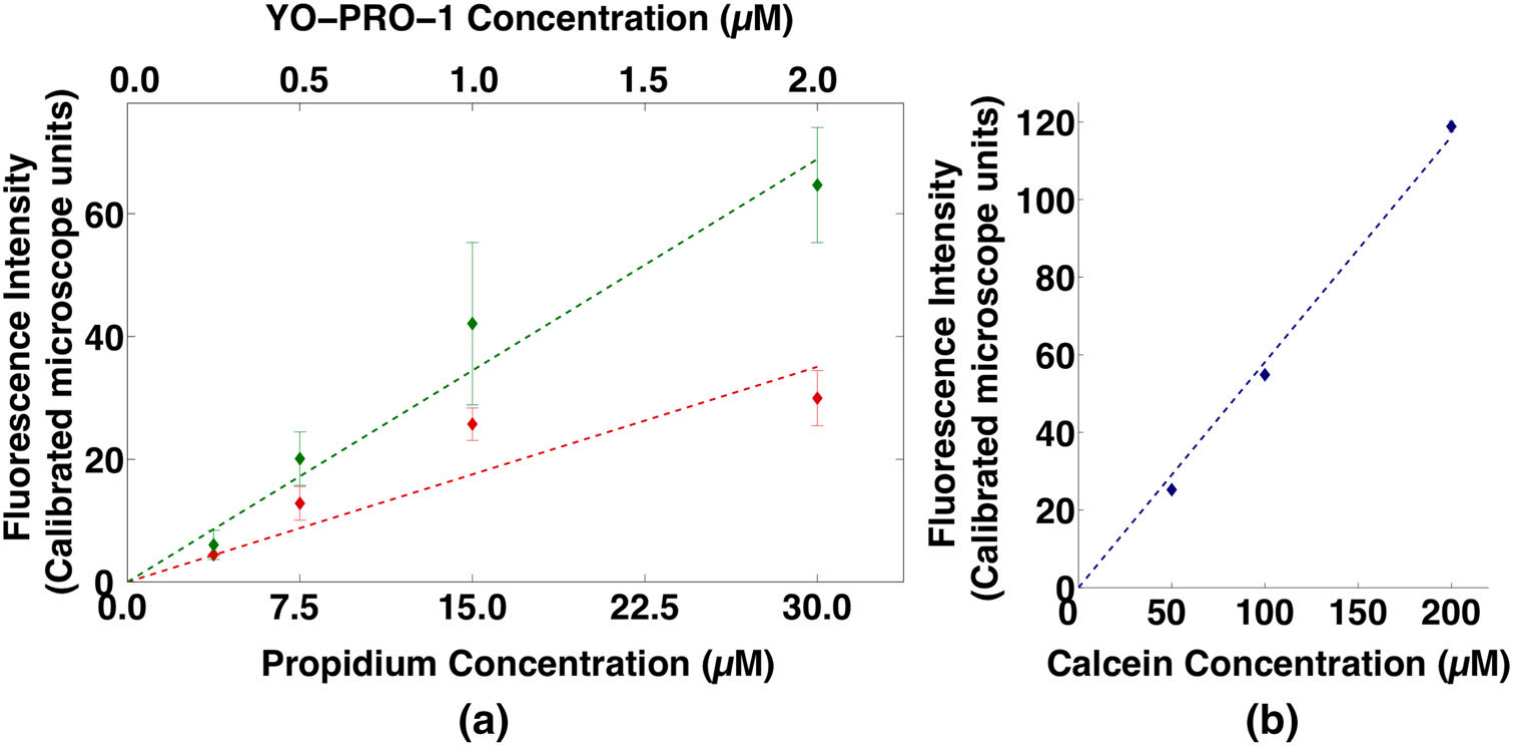
Calibration curves for **a** YO-PRO-1 and propidium and **b** calcein with Leica TCS SP8 confocal microscope. *Error bars* are SD

The procedure for correlating propidium and YO-PRO-1 fluorescence to molar concentration closely follows a method previously described (Pakhomov et al. 2015). Dense lysates were created from U-937 cells (8 × 10^7^ cells mL^−1^) by adding 0.1% Triton X-100 and then sonicating for 2 min with a Misonix Sonicator S-4000, 1 s alternating on–off cycle, amplitude 20. Calibration curves were generated by adding known concentrations of the dye to the lysate (Fig. 3a). Each point on the curves represents measurements taken in triplicate from three separate preparations. For calcein, the fluorescence of the extracellular medium (200 µM calcein) in each experiment was measured in three cell-free regions, and the mean of these measurements was used as the fluorescence intensity of 200 µM calcein. Measurements for lower calcein concentrations were taken in cell-free preparations (Fig. 3b).

## Results

### Transport Patterns After Microsecond Pulse Electropermeabilization

**Fig. 4:**
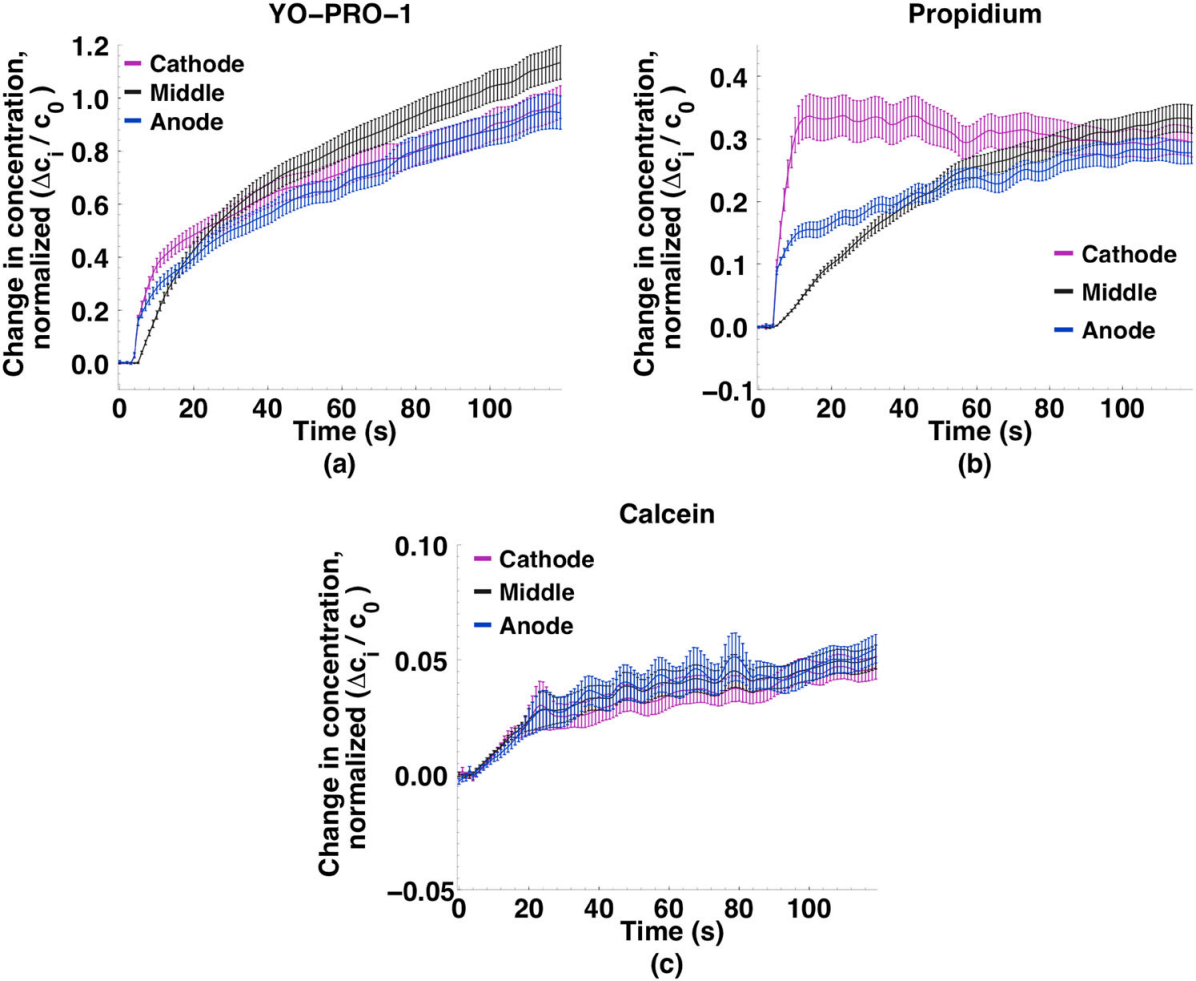
Small molecule concentration changes in cathode, middle, and anode sections of cells exposed to a single 220 µs, 250 kV m^−1^ pulse. **a** YO-PRO-1 (*n* = 30); **b** propidium (*n* = 34); and **c** calcein (*n* = 30). YO-PRO-1 and propidium entry into the cell is asymmetrical and quickly cathode-dominant; calcein enters equally from every direction. Data points are at 1 s intervals (300 ms in *inset*). Curves smoothed as described in methods

Figure 4 shows YO-PRO-1 (YP1), propidium (Pr), and calcein concentration changes (extracted from calibrated fluorescence images) in the anode- and cathode-facing and central regions of the cells after permeabilization by a 220 µs, 250 kV m^−1^ pulse. Within seconds after an initial burst of uptake at *both* the anode- and cathode-facing poles of the cells, *cathode*-side entry becomes dominant. The kinetics of transport into permeabilized cells are quite different for YP1 and Pr after the first few seconds. For YP1 the initial concentration differences among the three regions of the cell are essentially eliminated within 20 s. The Pr concentration in the middle of the cell increases more slowly, equilibrating with the anode and cathode regions only after 60 s. This can be interpreted to indicate that YP1 diffuses through the cytoplasm and binds to polynucleotides more quickly than Pr.

**Fig. 5:**
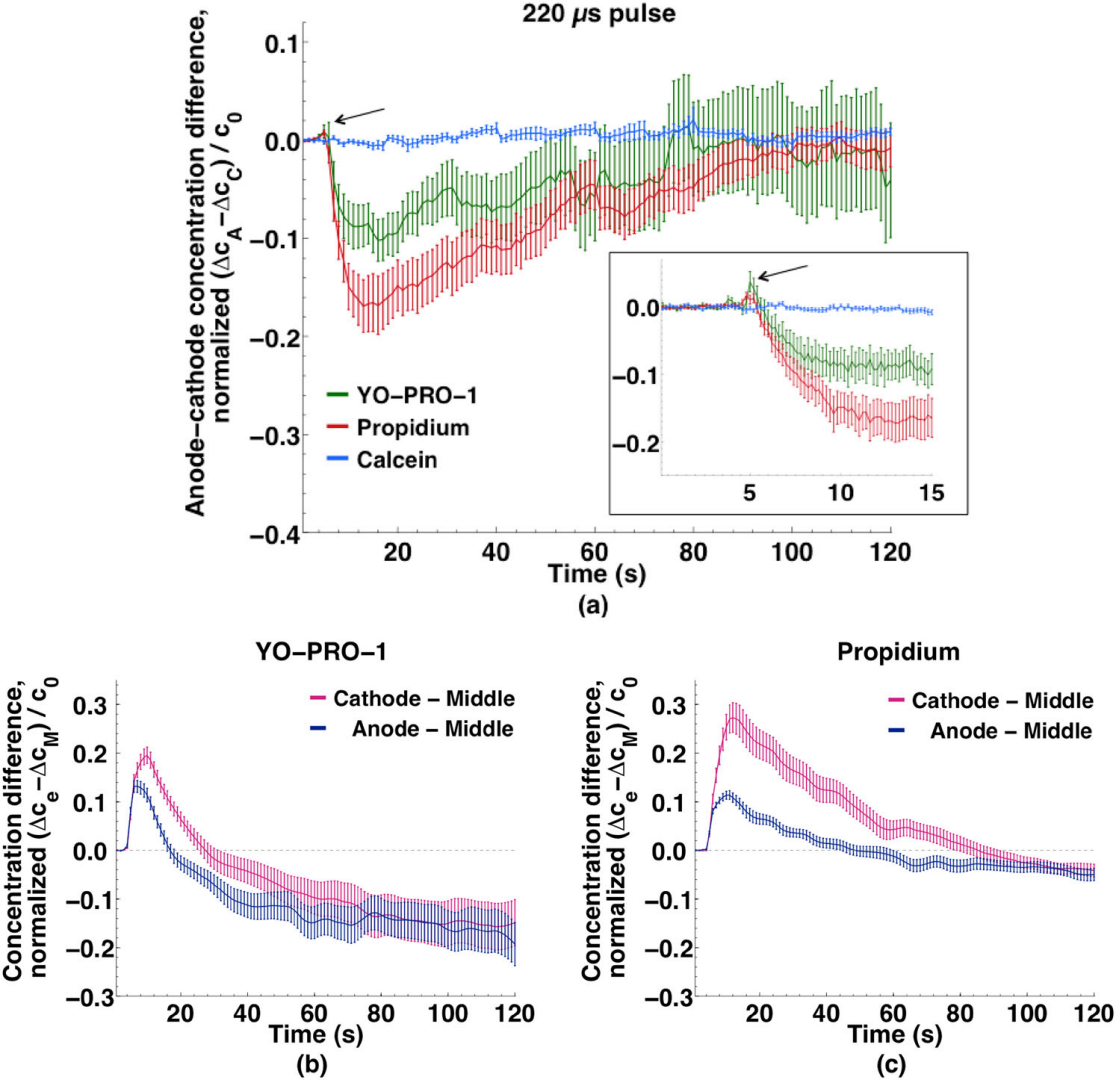
Localization of small molecule uptake in U-937 cells exposed to a single 220 µs, 250 kV m^−1^ pulse. **a** Uptake differences between anode and cathode regions of cells for YO-PRO-1, propidium, and calcein. Note the brief, initial, anode-side influx dominance that quickly switches to the cathode side. Decaying exponential functions fit to the data have time constants of 40 s for both YO-PRO-1 and propidium. No preferential uptake is observed for calcein. The early positive peak (*arrow*) indicates the initially greater anode-side influx switched to the cathode-side. Inset data points every 200 ms. **b** Differences in YO-PRO-1 uptake between anode and middle regions and cathode and middle regions of cells. After 25 s YO-PRO-1 concentration is greater in the middle region than in the anode or cathode regions. Curves smoothed as described in methods. **c** Differences in propidium uptake between anode and *middle regions* and cathode and *middle regions* of cells. Slower intracellular diffusion of propidium slows the decline of cathode and anode dominance. Curves smoothed as described in the methods

To highlight the symmetry or asymmetry in the entry patterns, the time course of the concentration differences between anode- and cathode-facing regions for all three dyes after delivery of a 220 µs pulse is shown in Fig. 5a. YP1 and Pr are clearly asymmetric (cathode-dominant), while no preferred region of entry is observed for calcein. Decaying exponential functions fit to the data have time constants of 40 s for both YP1 and Pr. Comparing the concentrations in the anode- and cathode-facing regions with those in the middle region of the cell for YP1 (Fig. 5b) and Pr (Fig. 5c) demonstrates the differences in uptake kinetics and intracellular distribution for these two molecules, each of which enters from both the anodic and the cathodic poles and migrates to the central regions of the cells. The YP1 concentration in the middle region exceeds the anode-region concentration after 15 s, cathode-region after 25 s. Propidium concentration in the middle of the cell increases more slowly, becoming greater than the anode- and cathode-region concentrations after 50 and 80 s, respectively.

**Fig. 6:**
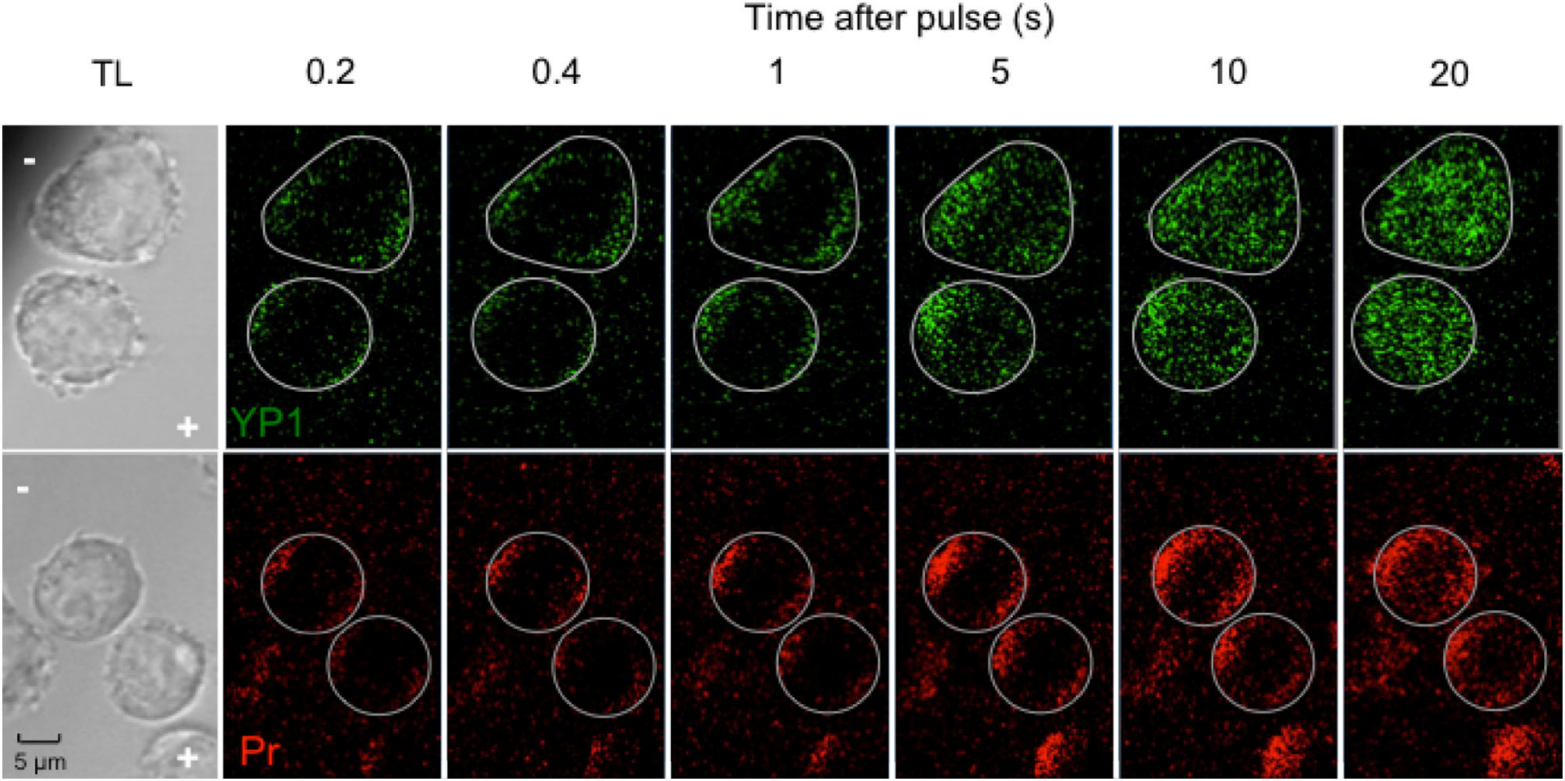
Fluorescence images of YO-PRO-1 and propidium transport into U-937 cells at 0.2, 0.4, 1, 5, 10, and 20 s after exposure to a single 220 µs, 250 kV m^−1^ pulse. *Circles mark* the circumference of the cells, which can be seen in the transmitted light images at the *left*, along with *plus* and *minus* symbols indicating the anode and cathode directions

The fluorescence images in Fig. 6 provide a visualization of YP1 and Pr entry into U-937 cells at several time points up to 20 s after delivery of a single 220 µs pulse. Cathode-side dominance is apparent for YP1 at 5 s, and sooner for Pr.

### Transport Patterns After Multiple Nanosecond Pulse Permeabilization

**Fig. 7:**
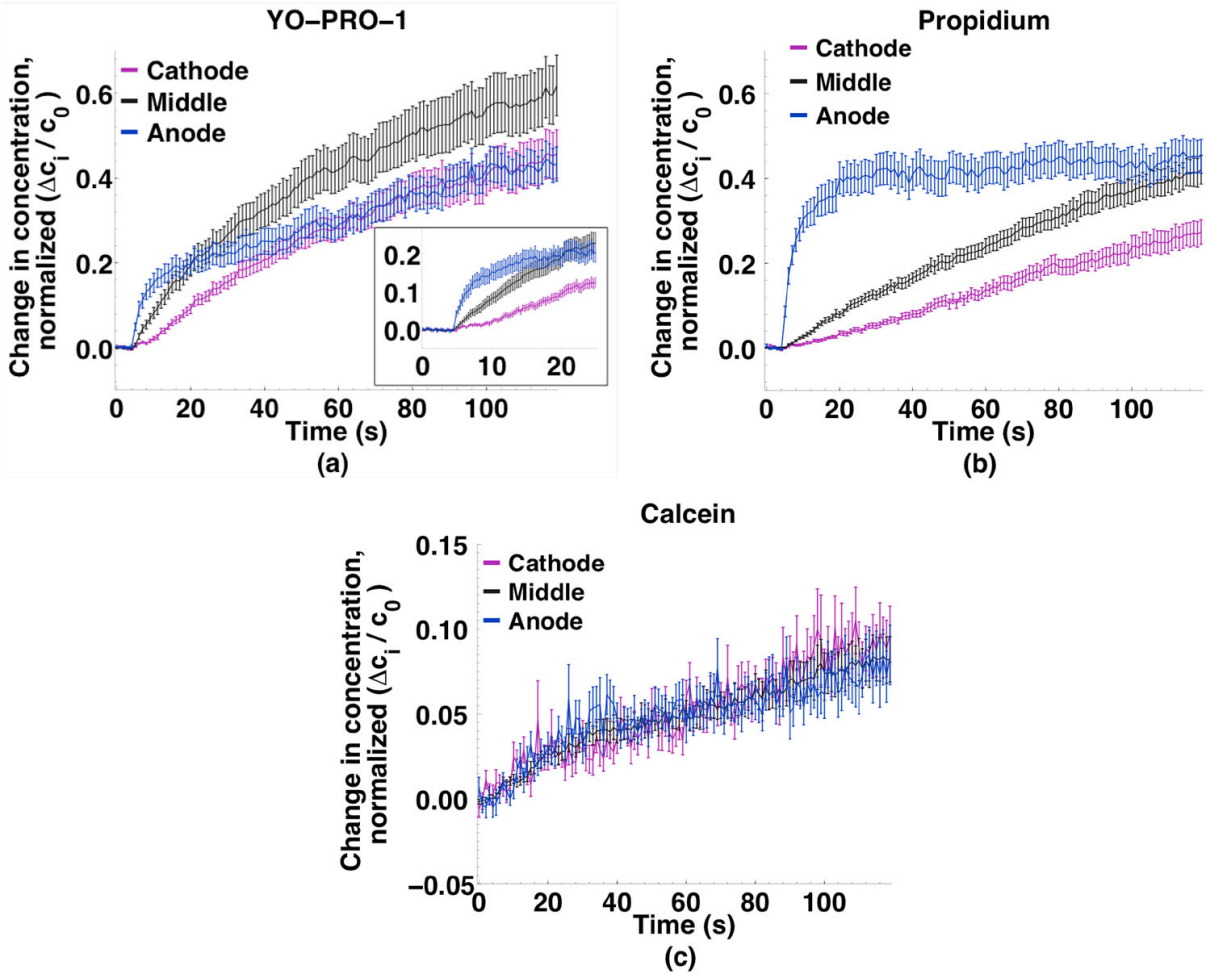
Small molecule concentration changes in cathode, middle, and anode sections of cells exposed to 10, 6 ns, 20 MV m^−1^ pulses at 1 kHz. **a** YO-PRO-1 (*n* = 34); **b** propidium (*n* = 30); and **c** calcein (*n* = 30). YO-PRO-1 and propidium entry into the cell is asymmetrical and initially *anode*-dominant; calcein enters equally from every direction. Data points are at 1 s intervals (300 ms in *inset*)

Figure 7 shows YP1, Pr, and calcein concentration changes in the anode- and cathode-facing regions and in the center of the cells after permeabilization by 10, 6 ns, 20 MV m^−1^ pulses delivered at 1 kHz. YP1 and Pr entry is again asymmetric, as it was after microsecond pulse permeabilization, but the pattern is quite different. YP1 and Pr increase immediately in the anode-facing region, then more slowly in the middle region, and still more slowly in the cathode-facing region, always anode-dominant, in contrast to the cathode-dominant influx observed for microsecond pulse exposures. The overall intracellular concentration of Pr increases more slowly than that of YP1; the cathode-middle-anode pattern for Pr over 120 s (Fig. 7b) resembles qualitatively the YP1 pattern over 20 s (Inset, Fig. 7a).

The amount of calcein entering permeabilized cells under these conditions is small compared to YP1 and Pr, and for calcein there is no preferential region of influx. For the microsecond and nanosecond pulse doses used in these experiments, the patterns of calcein uptake are similar.

**Fig. 8:**
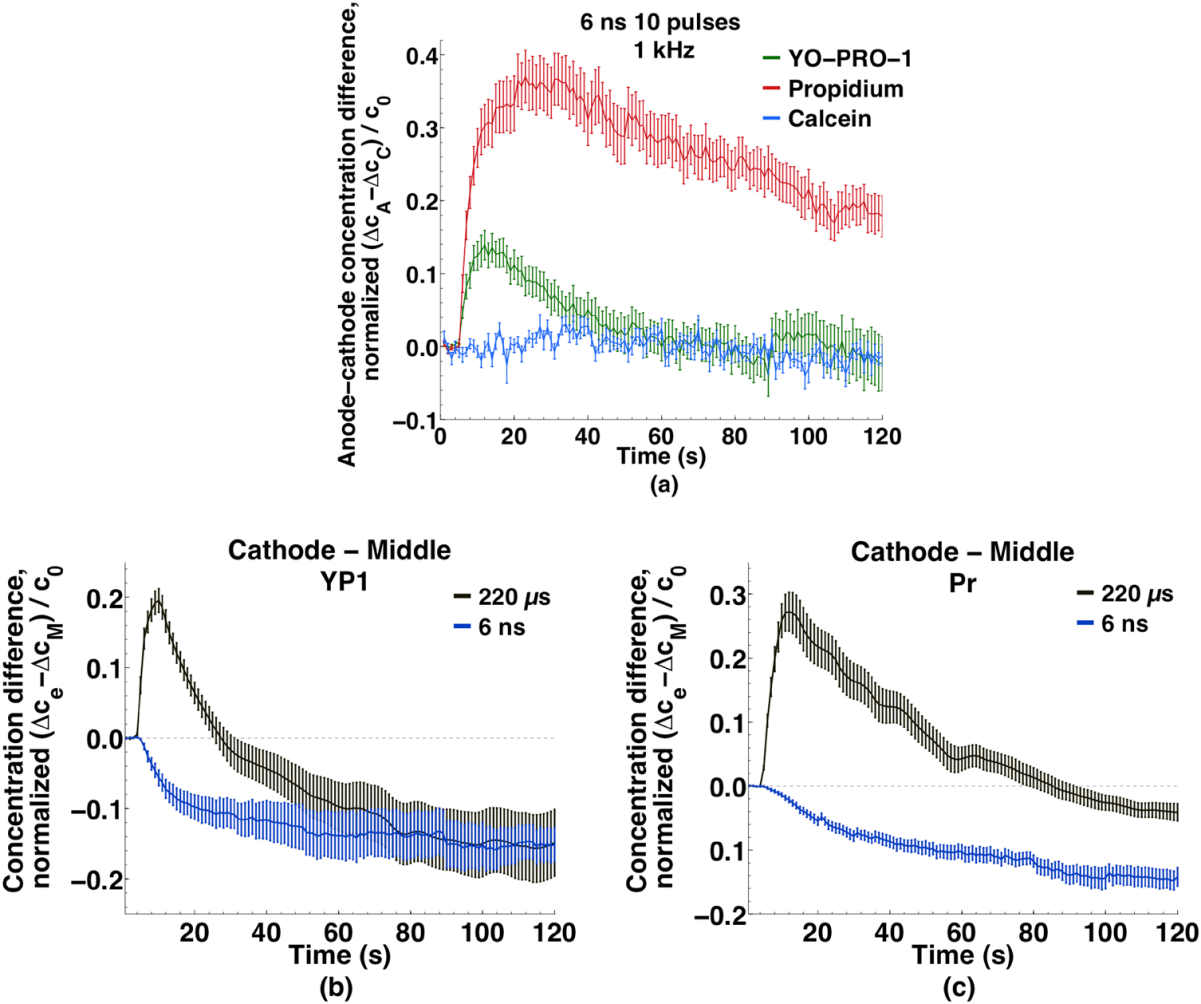
**a** Differences in small molecule uptake between anode and cathode regions of cells exposed to 10, 6 ns, 20 MV m^−1^ pulses at 1 kHz. Decaying exponential functions fit to the data have time constants of 20 and 140 s for YO-PRO-1 and propidium, respectively. **b** Relative absence of cathode-side influx for the nanosecond compared to microsecond pulse exposures for YO-PRO-1 and **c** propidium

To demonstrate more directly the uptake patterns for the multiple 6 ns pulse permeabilization observations, Fig. 8a shows the difference between concentrations in the anode- and cathode-facing regions over time for the three dyes. For YP1 and Pr, the large anode-side excess immediately after pulse delivery declines toward zero, more quickly for YP1 than for Pr. The uptake pattern is unidirectional, from the anodic poles of the cells toward the cathodic poles, again in contrast to the microsecond pulse case where the pattern was bidirectional (cathode-dominant, but with significant anode-side influx).

Note that the slower evolution of the *intracellular* Pr concentration gradient, which we associate with slower intracellular diffusion of Pr, produces an apparent difference in the asymmetry of *entry* between YP1 and Pr, if one looks only at times after 30 s. The total amount of YP1 and Pr transported into cells under these conditions, normalized against the extracellular concentration, is actually quite similar (this is the subject of a separate report).

The decay time constants for the exponential functions extracted from this data are 20 s for YP1 and 140 s for Pr. Calcein uptake in the two regions remains very similar during the 2 min recording period.

**Fig. 9:**
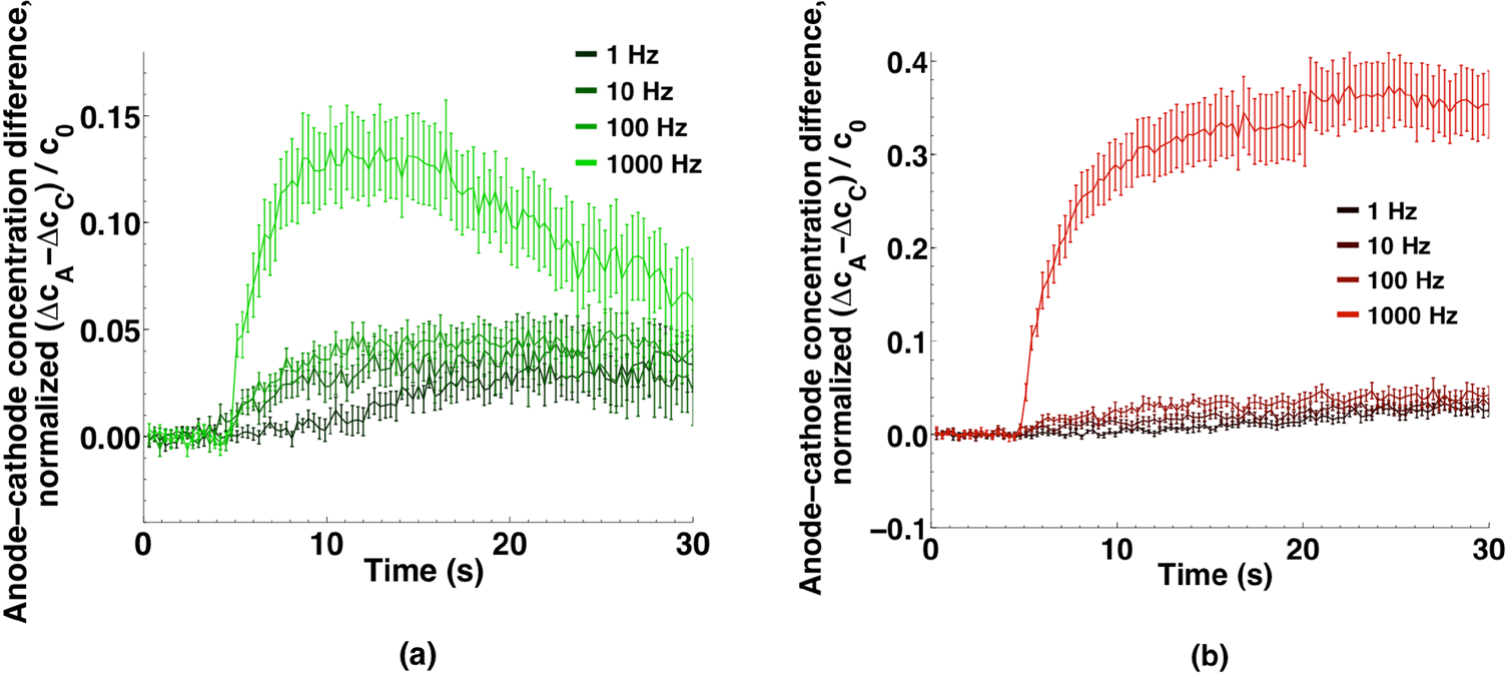
**a** YO-PRO-1 and **b** propidium transport asymmetry at different 6 ns nanosecond pulse repetition rates. (Note: pulse delivery begins at 5 s.) Anode-side dominance is sharply greater at 1 kHz, but still apparent over time even at 1 Hz. Data points are at 300 ms intervals

Figure 9 shows that anode-dominant entry can be observed for YP1 and Pr with 6 ns pulses at repetition rates from 1 Hz to 1 kHz. (At 1 Hz the asymmetry becomes detectable after the delivery of the fifth pulse (10 s into the recording). The markedly stronger response at 1 kHz for both YP1 and Pr may be an indicator of the time scale of the underlying permeabilizing processes. This direct dependence of membrane permeabilization on pulse repetition rate is consistent with our earlier reports where pulse durations were less than 10 ns (Vernier et al. 2006a, b, c; Romeo et al. 2013), but several studies using longer pulse widths see an apparently opposite trend (less permeabilization at higher repetition rates (Pakhomova et al. 2011, Silve et al. 2014) or mixed results (Steelman et al. 2016).

**Fig. 10:**
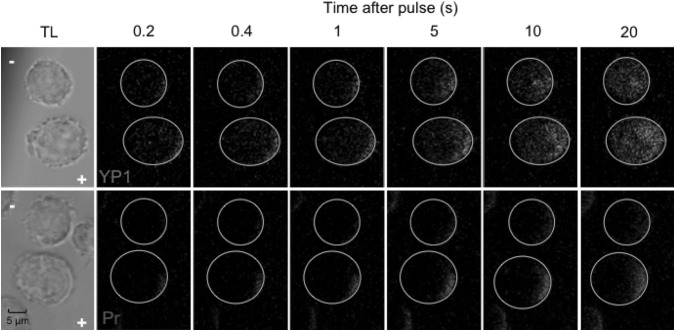
Fluorescence images of YO-PRO-1 and propidium transport into U-937 cells at 0.2, 0.4, 1, 5, 10, and 20 s after exposure to 10, 6 ns, 20 MV m^−1^ pulses delivered at 1 kHz. *Circles mark* the circumference of the cells, which can be seen in the transmitted light images at the *left*, along with *plus* and *minus* symbols indicating the anode and cathode directions

The fluorescence images in Fig. 10 show anode-dominant YP1 and Pr entry into U-937 cells at several time points up to 20 s after delivery of 10, 6 ns pulses at 1 kHz. YP1 diffuses through the cell much faster than Pr.

### YO-PRO-1 Transport Pattern After Single Nanosecond Pulse Permeabilization

No preferred direction of YP1 uptake was observed in initial observations with single-pulse 6 ns exposures, but since the fluorescence changes in those experiments are near the detection threshold in our system, it is possible that small anode-middle-cathode differences cannot be distinguished from noise in that data. To determine whether YP1 uptake after a single 6 ns permeabilizing pulse is distributed equally around the cell, as some models predict (Gowrishankar et al. 2006, Son et al. 2014), in contrast to the anode-side dominated influx observed after 10, 6 ns pulses, we repeated the single-pulse experiment with 10 µM YP1 in the extracellular medium instead of 2 µM YP1, to increase the level of YP1 fluorescence after permeabilization-induced YP1 influx. All other experimental conditions and imaging settings were the same.

**Fig. 11:**
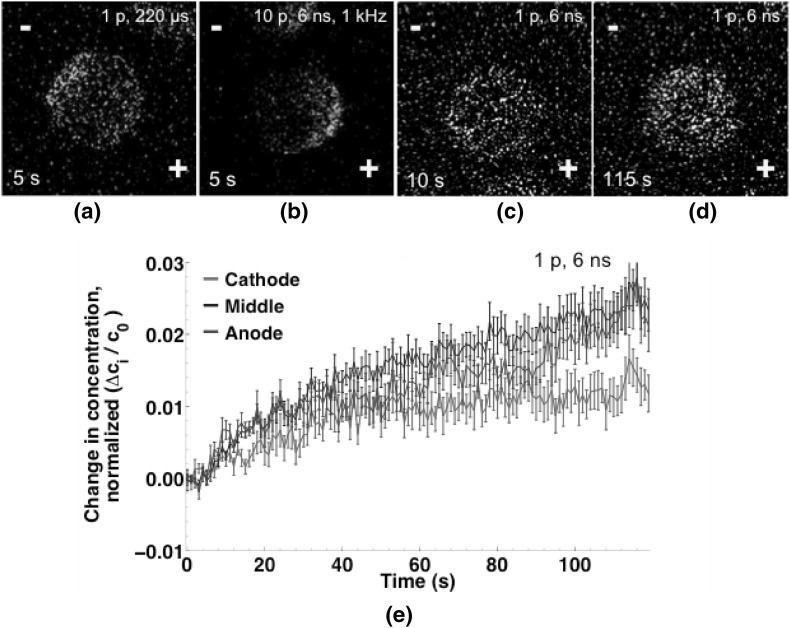
Fluorescence images of YO-PRO-1 uptake after pulse delivery. **a** 5 s after a single 220 µs, 250 kV m^−1^ pulse; **b** 5 s after 10, 6 ns, 20 MV m^−1^ pulses at 1 kHz; **c** 10 s after a single 6 ns, 20 MV m^−1^ (**d**) 115 s after a single 6 ns, 20 MV m^−1^ pulse. Intensity in panels **c** and **d** is increased to equalize the relative brightness of the images in the four panels. **d** Time course of YO-PRO-1 uptake in the cathode, middle, and anode regions of the cells after a single 6 ns, 20 MV m^−1^ pulse (*n* = 12). Extracellular YO-PRO-1 concentration for panels **c**, **d**, and **e** is 10 µM

With the stronger fluorescence signal resulting from the higher YP1 concentration, a slight asymmetry in the entry pattern can be measured (Fig. 11e), although it is not readily apparent from a visual inspection of the fluorescence images (Fig. 11c, d). Over 2 min, the YP1 fluorescence in the anode-facing and middle regions of the cells increases more than in the cathode-facing region, but the changes are gradual, unlike the immediate anode-dominant entry seen with the 10-pulse exposures. For comparison, the fluorescence images in Fig. 11 show the three YP1 entry patterns observed during this work: cathode-dominant (single 220 µs pulse), rapid anode-dominant (multiple 6 ns pulses), and slow anode-dominant (single 6 ns pulse). The calibrated, quantitative analysis of the data is presented in Fig. 11e.

## Discussion

### Calcein Transport is Always Symmetric

In contrast to YP1 and Pr, calcein transport into cells is symmetric around the cell with respect to the permeabilizing electric field, with no preferred direction of entry, for all of the experiments reported here. In fact, there is no published evidence of which we are aware for asymmetry in calcein transport into electropermeabilized cells under any conditions. If we can understand why calcein transport is always symmetric, we may gain some insight into the mechanisms for the asymmetry of YP1 and Pr transport.

YP1, Pr, and calcein are roughly the same size—respective molecular weights: 375, 414, 622; approximate cylindrical cross-sections (nm): 1.1, 1.4, and 1.2—and calcein’s cross-section is closer to YP1 than to Pr; so it seems unlikely that a sieving mechanism based on size is responsible for the apparent absence of asymmetric calcein transport.

One characteristic that distinguishes calcein from YP1 and Pr is its native fluorescence. Calcein is intensely fluorescent when dissolved in aqueous solution. YP1 and Pr fluorescence is relatively weak in aqueous solution but greatly enhanced when the molecules intercalate between stacked base pairs in a polynucleotide helical coil. When we observe YP1 or Pr entry into permeabilized cells, we are actually detecting the end of a multi-step process: transport into the cell, migration to an accessible nucleic acid molecule with helical structure, binding and intercalation between the polynucleotide base pairs.

In this context, asymmetric calcein entry could be undetectable at our image capture rate (200 ms^−1^). Since calcein is inherently fluorescent, the intracellular fluorescence pattern after calcein entry will be uniform as soon as calcein spreads throughout the cell. There will be no delay associated with polynucleotide binding or intercalation. The intracellular diffusion coefficient for calcein lies in the range 1 × 10^−10^ m^2^ s^−1^ (unrestricted volume) to 5 × 10^−12^ m^2^ s^−1^ (compartmentalized volume) (Brown et al. 1999). From the three-dimensional Einstein–Smoluchowski diffusion relation ($\langle {\text{x}}^{ 2} \rangle = { 6}Dt$), we can roughly approximate the time for calcein to diffuse across a free volume the size of a U-937 cell (*r* ≅ 12 µm) to be about 400 ms, or a compartmentalized volume (the actual intracellular environment is a combination of compartments and free volumes) in about 7 s.

Even if the calcein diffusion time was much shorter, a gradient of concentration from the entry point(s) on the membrane to the cell interior should be apparent as long as the intracellular concentration of the dye remains much less than the concentration in the external medium, and we see no plateau in the intracellular concentration of calcein in our data. So we consider this explanation for symmetric calcein entry to be unsatisfactory.

Another difference between YP1 and Pr on the one hand and calcein on the other is their electrical charge. In solution, YP1 and Pr are +2; calcein is −4. If the permeabilizing structures generated during exposure to pulsed electric fields are electrically polarized in a way that favors entry of cations, then calcein entry would be inhibited relative to YP1 and Pr. Indeed, the amounts of calcein transported into electropermeabilized cells are much less than the levels observed for YP1 and Pr, in our experiments and in the literature, but this does not provide an obvious mechanism for a geometric asymmetry of YP1 and Pr entry (anode- or cathode-facing pole of the cell) that would not result also in asymmetric entry for calcein, albeit at lower levels because of the hypothetical structural inhibition of anion entry.

Finally, if we assume that the entry of small molecules into electropermeabilized cells is not simply free diffusion through “holes” (pores) (Sabri et al. 1996) and that it involves also interactions of the impermeant solutes with the membrane, then it is reasonable to expect that entry patterns for calcein (tetravalent anion) may be different from those observed for YP1 and Pr (divalent cations). For example, attractive forces between YP1 and Pr and the anionic phospholipid phosphatidylserine (PS) will be stronger when PS (normally confined to the cytoplasmic leaflet of the plasma membrane) is externalized by electropermeabilization (Vernier et al. 2004a), and PS externalization occurs at the poles of the cells (Vernier et al. 2004b, 2006c; Escoffre et al. 2014). Calcein will not experience this attraction.

### Microsecond Pulse-Induced Uptake Patterns

The literature contains conflicting reports on patterns of small molecule uptake after long pulses (≫1 µs) like those typically used in electroporation applications. Microsecond pulse transport patterns for YP1 have not previously been reported. *Anode*-dominant uptake has been reported for ethidium (a slightly smaller (294 Da) analog of Pr) and Ca^2+^ with NIH 3T3 fibroblasts and Chinese hamster ovary (CHO) cells (Tekle et al. 1990; Tekle et al. 1994), and for neutral red (288 Da) with plant protoplasts (Mehrle et al. 1985, 1989). *Cathode*-dominant influx was observed for RH-160 (470 Da) with sea urchin eggs (Kinosita et al. 1992) and for Pr (414 Da) and ethidium homodimer (856 Da) with CHO, NIH 3T3, and HeLa cells (Tekle et al. 1994).

No simple electro-physical mechanism that might account for these disparate results, including our own observations of briefly *anode*-, then *cathode*-dominant influx for YP1 and Pr reported here, has been proposed. Predictions based on models of the magnitude and distribution of the transmembrane potential induced by an externally applied electric field, summed with the membrane resting potential (Tekle et al. 1990), even versions of those models in which the anode- and cathode-side distributions of pore size and area density are different (Saulis 1993, Krassowska and Filev 2007), cannot explain cases like those described above where the electropermeabilization-facilitated transport of a particular small molecule like YP1 or Pr can be anode-dominant in some cases and cathode-dominant in others, although they do provide plausible mechanisms for cathode-dominant influx for microsecond pulses. Similarly, electro-osmotic processes (Dimitrov and Sowers 1990) alone cannot account for transport that is anode-dominant in one case and cathode-dominant in another, *for the same solute*.

Considered carefully, our observations of microsecond pulse-induced Pr transport (initial anode-side dominance switching to cathode-side within seconds) are consistent with prior reports of Pr uptake patterns following “long” pulse exposures (Tekle et al. 1994; Gabriel and Teissié 1997, 1999). Note that the Gabriel and Teissié (1997, 1999) anode-dominant pattern data are observed for very short times (tens of milliseconds) after permeabilization, corresponding to the anode-side peak in Fig. 5a, and with much longer pulses (1–20 ms) than ours, making some differences in kinetics and pattern intensities not surprising. Additionally, Gabriel and Teissié (1997) report that slightly more than 50% of cells do exhibit cathode-dominant Pr fluorescence 30 s after permeabilization. In both reports, Gabriel and Teissié make the point that what they are measuring is Pr fluorescence, which may arise as much from interactions with membrane constituents as from intercalation in polynucleotides in the cell interior.

### Multiple Nanosecond Pulse-Induced Uptake Patterns

Our observations extend and quantify a previous report of anode-dominant transport for YP1 following multiple 4 ns permeabilizing pulse exposures (Vernier et al. 2006a, b, c) and another report for much longer, but still sub-microsecond, 600 ns pulses (Bowman et al. 2010). These are the only published descriptions of YP1 or Pr transport patterns following nanosecond electropermeabilization. Because the simple electro-physical model prediction that transport of small molecules will be greater on the anode side of the cell than on the cathode side (summation of induced membrane potential and resting membrane potential) fails for the long-pulse results above, we consider other mechanisms that could be responsible, at least in part, for the anode-side uptake of YP1 and Pr observed after 10, 6 ns permeabilizing pulses.

An explanation of small molecule uptake patterns will almost certainly include transport mechanisms that fall outside the classical electroporation model of diffusion through membrane pores. It is true that short-term (microseconds) transport of molecules like YP1 and Pr through electropermeabilized membranes might largely be accounted for by drift and diffusion through lipid pores, and classical electroporation models will be useful for analysis of this component of the overall process. However, experiments with artificial membranes and lipid vesicles indicate that lipid electropores are transient (lifetimes <10 ms; (Benz and Zimmermann 1981; Teissie and Tsong 1981; Melikov et al. 2001) and that most of the transport observed in studies like those reported here must take place either through pores that are modified in some way to hold them open, or through other structures in the electropermeabilized membrane.

Previous studies of nanosecond electropermeabilization reported pulse-induced phosphatidylserine (PS) externalization, a significant perturbation of the homeostatic membrane phospholipid bilayer configuration, primarily at the anode-facing side of the cell (Vernier et al. 2006a, b). Among other effects of this restructuring of the membrane, which involves at the same time formation of nanoscale lipid pores and the translocation of anionic phospholipids to the external leaflet of the lipid bilayer, we should not discount the change in the local electrostatic environment at the extracellular face of the lipid bilayer, which will be more negatively charged in regions where PS has been externalized. We noted above that this may tend to repel calcein (an anion), which could account in part for the uptake behavior observed for that molecule (and also the anionic Lucifer yellow). The negative charge may also attract the cations YP1 and Pr, facilitating the association of these molecules with the membrane interface more in regions of PS externalization (the anode pole of the cell) than elsewhere. These membrane–dye interactions can be expected to result in some fluorescence enhancement, as previously pointed out for Pr by Gabriel and Teissié (Gabriel and Teissié 1997), and they also may serve as anode-side sites of facilitated transport across the membrane.

Furthermore, it is not unreasonable to expect that the electropermeabilized membrane is disordered in ways that go beyond the formation of lipid pores and the scrambling of the lipid bilayer asymmetry associated with PS externalization. For example, nanosecond pulse-permeabilized cell membranes can be presumed to contain regions where lipid peroxidation products are present (Benov et al. 1994; Pakhomova et al. 2012), and because peroxidation of membrane lipids increases membrane permeability (Wong-ekkabut et al. 2007, Vernier et al. 2009), any tendency for the peroxidized regions to concentrate at the anode pole of the cell could contribute to anode-side asymmetry in YP1 and Pr entry.

Additional permeabilizing structures that could localize in the anode-facing region of the cell include associations or complexes of lipids and membrane proteins that are modulated by the bilayer scrambling and peroxidation activities mentioned above, or membrane proteins directly affected by the intense porating electric field (Chen et al. 1998, Gowrishankar et al. 1998). Finally, we can be sure that in response to all of these assaults on membrane integrity, cellular repair and restoration mechanisms are activated, which may result in temporary local increases in membrane permeability.

If formation of the initial population of lipid electropores and other permeabilizing structures is a function of the pulse-induced transmembrane voltage, as theory and experiment indicate, then we can expect that, like the transmembrane voltage itself, at least some of these downstream, long-term effects will be greater on the anode-facing side of the cell.

### Pulse Repetition Rate

The pulse repetition rate dependence of uptake for both YP1 and Pr after 6 ns pulse exposures, with markedly stronger responses at 1 kHz relative to lower rates, may be an indicator of permeabilizing processes with recovery times on the order of milliseconds. That is, when the time between pulses in a train is longer than the hypothetical recovery time, say 5 ms, physical and physiological membrane healing processes proceed to completion, or to some intermediate recovery state, and the membrane is affected similarly by each pulse. If the time between pulses is less than the recovery time, then each subsequent pulse is delivered with greater and greater effect to a more and more damaged membrane, in which the permeabilizing structures are either not repaired, or become stabilized. Although this simple hypothesis may be plausible for very short nanosecond exposures (Vernier et al. 2006a, b, c; Romeo et al. 2013), the picture becomes more complicated with longer pulses, where greater pulse repetition rates seem to be *less* effective (Pakhomova et al. 2011) or the situation is “complicated” (Silve et al. 2014, Steelman et al. 2016).

### Single Nanosecond Pulse-Induced Uptake Pattern for YO-PRO-1

YP1 uptake patterns for single and multiple (10) 6 ns pulse exposures are qualitatively different, reflecting more than simply an additive dose effect. There is no anode-side burst of uptake following a single pulse, and the distribution of the post-pulse intracellular YP1 fluorescence that develops gradually over 2 min is only weakly anode-dominant. If we accept the interpretation that some of the fluorescence patterns observed with both YP1 and Pr result from interactions of the dye molecules with the cell membrane (Gabriel and Teissié 1997), we can consider the possibility that a corresponding component of the molecular transport (a slower process involving smaller amounts of material) measured in our experiments is mediated through membrane-dye complexes, and some of the transport (faster, greater flux) involves more direct translocation through one or more of the permeabilizing structures mentioned above.

For the minimal permeabilizing dose represented by a single 6 ns pulse, relatively few of the faster permeabilizing structures would be created and stabilized, so the absolute amount of dye entering the cell is small, and the transport pattern is slow to develop and relatively symmetric. When multiple pulses are delivered, more of the faster permeabilizing structures are created, and for very short pulses they tend to accumulate on the anode side, for reasons described above. The result is the anode-dominant transport observed under those conditions.

### Intracellular Transport for YO-PRO-1 and Propidium

Why does YP1 seem to migrate through the intracellular volume 3–5 times faster than Pr (Figs. 5b, c, 8a)? In the simplest case where: (1) each dye enters at a single point on the cell membrane; (2). there is no significant binding of the dye to the membrane interface or to any intracellular material; and (3) the cell interior is a homogeneous fluid, the time it takes for each dye to reach any point inside the cell is proportional to the diffusion constant, about 4.2 × 10^−10^ m^2^ s^−1^ for YP1 and 3.2 × 10^−10^ m^2^ s^−1^ for Pr calculated based on their geometry (See Supplemental Material). By this measure, YP1 should be only 1.3 times faster than Pr1, so something else must be retarding the intracellular migration of Pr.

If we relax condition 2 above, and if Pr binds more tightly than YP1 to DNA, then its relative diffusion velocity through the cytoplasm will be reduced by encounters with polynucleotides. The dissociation constants of the two dyes with DNA, however, are similar (Biebricher et al. 2015, Wilson et al. 1985). Finally, relaxing condition 2 again, if Pr binds more tightly than YP1 to membranes, either the plasma membrane or the membranes of the endoplasmic reticulum and other organelles, then Pr will move more slowly through the cell, but we found no values for Pr-membrane or YP1-membrane binding constants.

## Concluding Remarks

Uptake patterns for three small molecule fluorescent indicators of membrane permeabilization following exposure of U-937 lymphoid cells in suspension to nanosecond and microsecond permeabilizing electric pulses demonstrate the still poorly understood complexity of electroporative transport. YO-PRO-1 (YP1) and propidium (Pr) entry after a microsecond (220 µs) pulse is concentrated initially at both anode- and cathode-facing sides of the cell, with cathode-side uptake quickly becoming dominant. Nanosecond, multiple-pulse (10, 6 ns) permeabilization results in primarily anode-side entry for YP1 and Pr, while a single nanosecond (6 ns) pulse produces a slow-developing (seconds), almost symmetric (slightly anode-dominant) YP1 uptake pattern. Calcein entry is symmetric for all of these pulse regimens, and the quantity of calcein entering electropermeabilized cells is much less than that for YP1 or Pr for similar pulse doses.

These results, which are not predicted or explained by current models and schemes of electroporation, suggest that electropermeabilization-induced transport of small molecules is dependent in each case on the properties of the solute species, and that, indeed, more than simply concentration-driven diffusion through holes in the cell membrane is involved (Teissié et al. 1999).

The anode- and cathode-side uptake patterns we observe, considered together with previously published theoretical and experimental reports, cannot arise simply from the asymmetry of transmembrane potential during pulse delivery. We suggest that a satisfactory explanation will include the contributions of induced and resting transmembrane electrical potentials, electropore formation kinetics, pore size and area distributions, pulse-induced membrane disorganization (transbilayer and intra- and inter-domain scrambling), anionic phospholipid externalization, and interactions between the membrane and the normally impermeant solute.

A hypothetical scenario might look like the following. Variations in local transmembrane potential (the sum of the resting potential and the position-angle-dependent potential induced by the external pulsed electric field) over the surface of the cell result in an asymmetric distribution of short-lived lipid electropores that are associated also with sites of PS externalization and the formation of membrane-solute aggregates. A long (microsecond) pulse expands the size of the pores, preferentially on the cathode side of the cell. Short-term transport (<1 s) takes place primarily through the short-lived electropores created during the application of the external electric field. Long-term transport results from downstream processes that are initiated by or enhanced by the permeabilizing pulse, such as PS externalization, disordering of membrane bilayer asymmetry and domain organization, and membrane-solute interactions. In this scheme, long-term transport is cathode-dominant for microsecond pulse poration because of the influx through the expanded pores on the cathode-facing side of the cell, and anode-dominant for nanosecond pulses, because of the greater anode-side membrane disorganization driven by anionic lipid translocation.

## Electronic Supplementary Material

Below is the link to the electronic supplementary material.

Supplementary material 1 (DOCX 96 kb)
