# Supplementary material for: Asymmetric Patterns of Small Molecule Transport After Nanosecond and Microsecond Electropermeabilization
Source: J Membr Biol. 2017 May 8;251(2):197–210. doi: 10.1007/s00232-017-9962-1 (PMC5910485; doi:10.1007/s00232-017-9962-1)
Supplement: Supplementary file 1 — Supplementary material 1 (DOCX 96 kb) [file 232_2017_9962_MOESM1_ESM.docx]

# Supplementary Material

**Asymmetric Patterns of Small Molecule Transport After Nanosecond and Microsecond Electropermeabilization**

Esin B. Sözer, C. Florencia Pocetti, P. Thomas Vernier

## Diffusion Coefficient Estimates for YO-PRO-1 and Propidium

Values for the diffusion coefficients of YO-PRO-1 (YP1) and propidium (Pr) are estimated based on the geometrical properties of the molecules as described in Smith **(2011)**. For a spherical particle with a well-established diffusion coefficient we used the sodium ion as a reference. From its diffusion coefficient (*D_Na_*), and radius (*r_Na_*), we can extract an estimate for the diffusion coefficient of an ellipsoid particle with longer radius (*l_s_*/2) and shorter radius (*r_s_*).

Diffusion coefficient (*D*) and friction coefficient (*ξ*) are related **(Dill and Bromberg 2010)**:

$D=\frac{\text{k}T}{\xi}$ (S1)

where k are Boltzmann’s constant, and absolute temperature. For a spherical particle like the sodium ion the friction coefficient *ξ* is

$\xi_{Na} = 6\pi\eta r_{Na}$ (S2)

where *η* is the viscosity of the solvent. For ellipsoid solutes moving randomly, with longer radius (*l*_s_/2) and shorter radius (*r*_s_), the friction coefficient is

$\xi_{\text{s}}=\frac{6\pi\eta(l_{\text{s}}/2)}{\ln(l_{\text{s}}/r_{\text{s}})}$ (S3)

Using S1, S2, and S3, we can get diffusion coefficient for a solute *s*:

$D_{\text{s}}=\frac{D_{\text{Na}} r_{\text{Na}}}{l_{\text{s}}/2}\times\ln\left(\frac{l_{\text{s}}}{r_{\text{s}}}\right)$ (S4)

From S4, with *D_Na_* = 1.33 × 10^-9^ m^2^ ⋅ s **(Hille 2001)** and *r_Na_* = 0.23 nm, the diffusion coefficient at 25° C for YP1 is 4.2 × 10^-10^ m^2^ ⋅ s and for Pr is 3.2 × 10^-10^ m^2^ ⋅ s.

Geometrical parameters used are *l_YP1_* = 1.71 nm, *r_YP1_* = 0.53 nm, *l_Pr_* = 1.55 nm, *r_Pr_* = 0.69 nm **(Smith 2011)**.

## References

Dill K, Bromberg S (2010) Molecular driving forces: statistical thermodynamics in biology, chemistry, physics, and nanoscience. Garland Science

Hille B (2001) Ion channels of excitable membranes. Sinauer Sunderland, MA

Smith KC (2011) A Unified Model of Electroporation and Molecular Transport. Thesis, Massachusetts Institute of Technology.
